# IFN Signaling in Inflammation and Viral Infections: New Insights from Fish Models

**DOI:** 10.3390/v11030302

**Published:** 2019-03-26

**Authors:** Christelle Langevin, Pierre Boudinot, Bertrand Collet

**Affiliations:** INRA, Virologie et Immunologie Moléculaires, Université Paris-Saclay, 78352 Jouy-en-Josas, France; Christelle.Langevin@inra.fr (C.L.); Bertrand.Collet@inra.fr (B.C.)

**Keywords:** fish, type I interferon, vaccine, adjuvant, hematopoiesis, genome editing, Crispr, innate immunity

## Abstract

The overarching structure of the type I interferon (IFN) system is conserved across vertebrates. However, the variable numbers of whole genome duplication events during fish evolution offer opportunities for the expansion, diversification, and new functionalization of the genes that are involved in antiviral immunity. In this review, we examine how fish models provide new insights about the implication of virus-driven inflammation in immunity and hematopoiesis. Mechanisms that have been discovered in fish, such as the strong adjuvant effect of type I IFN that is used with DNA vaccination, constitute good models to understand how virus-induced inflammatory mechanisms can interfere with adaptive responses. We also comment on new discoveries regarding the role of pathogen-induced inflammation in the development and guidance of hematopoietic stem cells in zebrafish. These findings raise issues about the potential interferences of viral infections with the establishment of the immune system. Finally, the recent development of genome editing provides new opportunities to dissect the roles of the key players involved in the antiviral response in fish, hence enhancing the power of comparative approaches.

## 1. Introduction

Most of the basic features of the immunity processes are conserved across vertebrates, but the anatomical and physiological fundamental differences between fish and tetrapods make comparative analysis interesting, as it reveals many specific adaptations and original mechanisms.

Type I and type II interferons (IFN) have been described in fish [1,2,3]. While structural studies have definitely demonstrated that fish type I IFN encoded by intron-containing genes are the true counterparts of mammalian type I IFN [4], these cytokines have diversified independently during fish and tetrapod evolution [5]. Type I IFN is highly variable across fish species, with zebrafish laboratory strains having only four genes coding type I IFN (named IFNφ) and salmonids having over 30 type I IFN genes [6]. These IFN are classified into two groups, depending on the number of cysteine bridges present in the protein, and further divided into subtypes based on sequence similarity [7]. For example, in Atlantic salmon, six subtypes have been distinguished (a–e): IFNa, d, and e have two cysteines and belong to group 1, while IFNb, c, and f have four cysteines and belong to group 2 [6]. Even in fish species in which type I IFN are relatively well described such as Atlantic salmon, rainbow trout, and zebrafish, the specific functions of genes or subtypes remain elusive. In Atlantic salmon, Robertsen et al. have shown that IFNa1 (group 1) and IFNc (group 2) had strong antiviral activity against IPNV in vitro, while IFNb (group 2) was less potent, and IFNd (group 1) was not active at all [8]. While type I IFN receptors have been identified in zebrafish and salmonids, the detailed structure of signaling pathways within the fish IFN remain poorly characterized, and is mainly inferred from what is known in mammals.

Most functional studies of IFN antiviral activity have been performed in vitro using fish cell lines, and only a few studies have compared different type I IFN in vivo. In one example of such studies performed in zebrafish, Lopez-Munoz reported that while IFNs from both group 1 (IFNφ1) and group 2 (IFNφ2 and φ3) have antiviral activity in vivo, only IFNφ1 provides efficient protection against the fish bacterial pathogen *Streptococcus iniae*. This study also suggested different kinetics of induction of effector IFN-stimulated genes (ISG) by group 1 and 2 IFNs [9]. The in vivo characterization of type I interferon activity constitutes a major challenge to better understanding fish/virus interactions, identifying IFN producing cell subtypes, and obtaining an integrated view of the fish IFN contribution to antiviral defenses. In particular, the work performed on cell lines has completely overlooked the role of IFN in the interaction between innate and adaptive antiviral responses, as well as all its potential functions in development, cell differentiation, and regulation of the cell cycle. A lot of in-depth research is still required to fully understand the regulation mechanisms and the functional properties of these type I IFNs across the great diversity of teleost fish.

The antiviral functions of fish type I IFNs and their mechanisms have been recently reviewed [5,10,11,12,13]. In this review, we focus on the new developments of three different topics that will likely be important in the fields of fish interferons in the coming years. We first discuss the role of type I IFN activity in the development of protection against viral diseases provided by DNA vaccination in fish. In a second section, we examine the role of sterile and pathogen-induced inflammation on the regulation of hematopoietic stem cell differentiation, and the potential impact of viruses on the maturation of immunity. We discuss the available data in zebrafish and the perspectives offered by this model. Finally, we present the recent development in genome editing, especially those based on CRISPR/cas9 technologies and their contribution to the understanding of antiviral mechanisms in fish.

## 2. Adjuvant Antiviral Activity of Fish Type I IFN

In fish, DNA vaccines against viral diseases are remarkably efficient when administered intramuscularly [14,15,16]. A local transient type I IFN induction shortly after the administration of a protective DNA vaccine is consistently observed, and may be correlated with long-term antigen-specific protection in fish [17,18,19]. A mechanism for such a correlation has been described in higher vertebrates whereby type I IFN response is induced by the detection of intracellular CpG-containing plasmid DNA by STING [20], and initiates the adaptive immunity through the action of dendritic cells (DC) [21,22]. More generally, many reports point to nucleic acid sensing pathways to explain type I IFN adjuvant effects: the activation of cGAS, RIG-I, and TLR7 has been used to trigger an IFN-dependent increase of vaccine efficacy [23,24,25]. As nucleic acid sensors, adaptors and VISA are generally well conserved across vertebrates [26,27,28], the use of helicases and other nucleic acid sensors appear promising for the development of new adjuvants in fish, especially for DNA vaccines.

A strong adjuvant capacity of fish type I IFN was first reported by Robertsen et al., who used a model of DNA vaccine against the infectious salmon anemia virus (ISAV), which is based on the hemagglutinin esterase (HE) of the orthomyxovirus infectious salmon anemia virus (ISAV) [29]. The vaccine was administered intramuscularly (i.m.) either alone or in combination with expression plasmids for IFNa1 (group 1), IFNb, or IFNc (group 2). Remarkably, while the vaccine alone led to a poor protection and low Ab titers, all three IFNs improved the protection against ISAV after a challenge performed 10 weeks post-vaccination. Additionally, serum titers of anti-ISAV Ab were also significantly increased. Interestingly, Sobhkhez et al. demonstrated that the over-expression of the ISAV HE in CHinook Salmon Embryo (CHSE) cells was able to suppress the IFNa1-induced mx1-promoter reporter activity [30]. Hence, the intrinsic ability of HE alone to protect against ISAV infection [29] could be affected by the IFN-suppressive property of this protein. Compensating this effect by providing a type I IFN-expressing plasmid may restore a high level of protection. Unfortunately, the IFN activity shortly after the administration of the HE DNA vaccine alone was not measured [29]. A generic adjuvant effect of type I IFN will have to be evaluated in other models in which the antigen does not suppress the IFN response.

**Figure 1:**
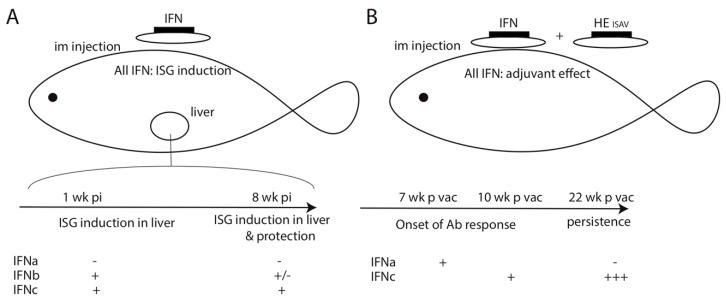
Effects of injection of plasmids encoding different type I interferon (IFN) on salmon susceptibility to virus and on vaccine efficacy when administered alone or in combination with a DNA vaccine to infectious salmon anemia virus (ISAV). (**A**). Injection of plasmids encoding type I IFN leads to contrasted levels of ISG induction in distant organs, and of protection eight weeks post-injection. (**B**). The co-injection of all plasmids encoding different type I IFN (a, b, and c) and of the DNA vaccine encoding ISAV hemagglutinin esterase (HE) protein affords adjuvant effect; however, the onset of Antibody (Ab)response and its persistence are higher for IFNc.

These adjuvant effects of IFNa1, b, and c were in contrast with their very different direct antiviral activities in vivo, as reported by the same group after the i.m. injection of expression plasmids [31] (Figure 1). While all three plasmids induced ISGs locally at the site of injection, they differ in their capacity to mediate long-range effects. One week after injection, IFNc and b—but not a—led to ISG up-regulation in distant organs such as pronephros and liver. Strikingly, the i.m. injection of IFNc plasmid mediated a long-term (up to eight weeks) modulation of ISG expression in pronephros, and a strong protection against a challenge with ISAV eight weeks post-injection. Taken together, these data suggest that the adjuvant activity of the type I IFN relied on the local expression of IFNa, b, or c rather than on the distant activation of antiviral pathways. First insights from the RT-PCR quantification of Ig, CD3, CD8, perforin, and IFNγ mRNAs suggested that the adjuvant effect was associated with a recruitment of B-cells and T-cells close to the injection site [29], which was likely triggered by IFN-induced local inflammation.

Differences among the adjuvant activity of IFNa, b, and c were observed when Ab responses were studied over longer periods [32]: after the co-injection of the vaccine and IFNa plasmid, the Ab response against ISAV developed at seven weeks post-vaccination, while with the IFNc plasmid, the response was observed later, 10 weeks post-vaccination. The peak of Ab response was also shifted: it was 10 weeks for IFNa, and 16 weeks for IFNc. The Ab rates were sustained at a high level for at least 22 weeks post-vaccination in the case of IFNc adjuvating. Interestingly, the expression of ISG induced by IFNc persisted much longer when the expression plasmid was injected alone, compared to when it was administered in combination with the HE plasmid, which may fit with the anti-IFN effects of HE, as discussed above [30]. Alternatively, the authors proposed that transfected cells expressing the viral protein may be eliminated by the cytotoxic adaptive responses of the host, while cells expressing only IFNc were tolerated, since this protein was recognized as part of the host (i.e., as “self”). This elimination of IFNc-producing cells in the vaccination context is potentially important for the efficiency of the adjuvant effect. Indeed, adverse effects of type I IFN have been noted during persistent Lymphocytic ChorioMeningitis Virus LCMV infection in mice, where chronic IFN expression can polarize CD4 T cell responses toward T follicular helper (Tfh) and favor CD8 T cell exhaustion [33].

In another report, the adjuvant effects of the rainbow trout intracellular IFNa (iIFNa, [34]) on a DNA vaccine encoding the glycoprotein of infectious hematopoietic necrosis virus (IHNV) have also been reported [35]. Surprisingly, in this study, neutralizing Ab titers that were observed after i.m. injection with the IHNV G plasmid alone peaked at 21 days post-vaccination, and had already declined by day 35; meanwhile, the co-injection of the iIFNa plasmid maintained the neutralizing Ab titer at the peak level at this time point. Also in contrast to other reports (reviewed in [16]), vaccination with the vaccine alone provided only 50% protection when a challenge was performed 28 days post-vaccination. In this context, in line with the sustained neutralizing Ab rate, iIFNa plasmid seemed to increase fish survival. The mechanisms involved remain to be explored, and a comparison of the adjuvant effects of intracellular and secreted type I IFN will certainly be interesting, especially with viral challenges performed at later time points.

The adjuvant effect of the IFN-inducing factor DDX41 has also been reported for a DNA vaccine against VHSV in olive flounder [17]. Expression plasmids for the VHSV glycoprotein or for the helicase DDX41 alone were compared to a plasmid encoding both proteins under the control of EIF and Cytomegalovirus (CMV) promoters, respectively. Fish were challenged 15 or 30 days after vaccination, and the group injected with the plasmid coding for the G protein and DDX41 appeared to be better protected than those injected with the G vaccine alone. While these results show that the expression of DDX41 combined with the DNA vaccine-encoded antigen can modify the outcome of an early challenge, further experiments are needed to determine whether this effect is sustained for longer periods and is antigen-specific (i.e., by the absence of cross-protection against other viruses).

In mammals, it has been shown that type I IFNs are essential for the effects of a number of adjuvants, including for example, complete Freund adjuvant and chitosan [24,36]. In some cases, these mechanisms have been observed even if only DC can sense IFN, showing that these cells are required and sufficient for the adjuvant to take place [37]. Overall, observations on adjuvant effects in these models are consistent with mechanisms based on IFN-dependent immunoregulation rather than local enhancement of the innate antiviral response. The long-term protection of a number of DNA vaccines in fish is associated with seroconversion and the presence of neutralizing antibodies (such as infectious hematopoietic necrosis [IHN] in rainbow trout [38,39], viral hemorrhagic septicemia (VHS) [19], and pancreatic disease [40]). After intramuscular injection, myocytes located in the needle track expressed the antigen, as shown in DNA vaccination against VHS [41,42]. These cells are capable, at least in mammals, of expressing MHC class II [43]. However, it is not known whether fish myocytes are able to present the antigen directly to the immune system. Alternative mechanisms may involve the action of resident or circulating antigen-presenting cells. In this context, the ability of dendritic cells (DC) for antigen cross-presentation would provide some pertinent hypothesis to explain the high efficacy of DNA vaccination [44]. A number of studies have shown that type I IFNs help DC differentiation and activation in many mammalian models (reviewed in [45]). While the presence of DC in fish has been reported [46,47,48], still, no reliable cell surface markers or associated reagents are available to isolate DC from the commercial fish species where DNA vaccination studies were conducted. In fish, in the absence of lymph nodes and germinal centers, the mechanisms by which antigens are captured and interact with B and T lymphocytes remain unknown. The remarkable role of type I IFN in salmonids in the efficacy of antiviral vaccines constitutes a relevant model to explore this field. Further work will determine the respective importance of DC and other cell types expressing IFN receptors such as NK and B cells [49] in mediating IFN-dependent adjuvant effects. Finally, it is worth mentioning that the intramuscular injection of a small volume of material in salmonid fish, including DNA vaccine plasmid, results in local tissue damage and hemorrhage (BC, personal observation) potentially facilitating other systemic immune cell types to either interact with transfected cells and/or the plasmid to become transfected themselves. This fact is poorly documented, but indirectly evidenced by the detection of plasmid in the blood as early as one minute after intramuscular injection in rainbow trout [50], which is consistent with hemorrhage during DNA vaccine i.m. injection.

To date, DNA vaccines are in use in commercial fish farms in Canada (APEX-IHN®, Elanco) and in Europe (CLYNAV®, Elanco). Their combination with plasmids encoding type I IFN have the potential to further improve already efficient DNA vaccines by either increasing the longevity of protection or reducing costs. A better understanding of the mechanisms involved will be an important challenge for the fish virology and immunology in the coming years. With many type I IFN divergent subgroups [6], one can expect a large variation of functional properties and applicability to vaccination across different commercial fish species. This emphasizes the need for an in-depth characterization of the immunoregulatory mechanisms mediated by these key cytokines.

A number of important questions that are needed in order to understand the mechanism of long-term protection induced by DNA vaccines remain unanswered. (1) Which cells other than myocytes are expressing/presenting the antigen? (2) Is there any infiltration of circulating immune cells to the site of injection? (3) What is the role and mechanism of early and transient type I IFN induction? With the recent development of genome editing technology, the last question may now be addressed using type I IFN receptor knock-out in commercial fish models. However, this approach will be challenging in certain species in which the number of paralogs is significant. (4) DNA vaccines have been developed against fish DNA viruses [51,52]. These viruses are often able to induce type I IFN response, and have acquired genes of cytokines that may dysregulate the host immune response [53,54]. Therefore, it will be interesting to test the adjuvant effects of type I IFN in such cases, and compare their mechanisms with RNA viruses.

## 3. Inflammation and Hematopoietic Stem Cell Differentiation

Besides its antiviral and antiproliferative properties, type I IFN is also an important factor for immune cell differentiation [55]. IFN-dependent mechanisms of leukocyte differentiation may be activated by the inflammatory context of innate responses to infections. However, the maintenance of inflammation leads to tissue damage, and sometimes evolves to chronic inflammatory diseases, which also affect immune cell dynamics [56]. IFN impact on cell differentiation has also been described in sterile conditions, as part of the physiological differentiation program. The description of mechanisms sustaining hematopoiesis regulation by pro-inflammatory signaling is an active field of research, but many open questions remain, especially regarding the resilience mechanisms after infections. In this regard, the zebrafish embryo constitutes an interesting vertebrate model, which is based on the high similarity between the zebrafish and human antiviral innate immune response [57] and conserved hematopoiesis processes [58]. As in mammals, blood development occurs through primitive and definitive waves in zebrafish [59,60], which relies on conserved genetic pathways, even though hematopoietic organs are partly different from those of mammals [61,62]. Embryonic primitive hematopoiesis gives rise to primitive erythrocytes and primitive myeloid cells (neutrophils and macrophages) in the lateral mesoderm, which is the equivalent of the mammalian yolk sac before 24 hours post-fertilization (hpf). From 26 hpf, hematopoietic stem cells (HSC) emerge from the transition of dorsal aorta endothelium to hematopoietic cells during the definitive wave [63,64]. HSC then migrate to colonize caudal hematopoietic tissue, which is the equivalent of the mouse fetal liver and placenta, to give rise to erythrocytes, thrombocytes, and myeloid cells. Starting at 48 hpf, HSC colonize the pronephric marrow, which is the equivalent of mammalian bone marrow, and differentiate in all of the blood cell lineages: erythrocytes, thrombocytes myeloid cells, and lymphoid cells. From 54 hpf, lymphoid progenitors reach the thymus to differentiate in lymphocyte T cells, which colonize the pronephros and the rest of the body [65].

Advantages of the zebrafish model (optical transparency, genome editing tools, and the transgenic animals library) have been largely exploited to describe in real time the molecular and cellular processes occurring during hematopoiesis, combining microscopy on transgenic lines expressing fluorescent reporters specifically in HSC (runx1, cmyb), vascular endothelium (kdrl), and immune cell populations (mpeg, mpx) [66,67]. Gain and loss of function experiments based on transgenesis, mRNA, or morpholinos injection [68] have been instrumental to identifying the genetic factors involved in the emergence of HSC from the hemogenic endothelium in the dorsal aorta, as well as HSC specification, proliferation, and/or differentiation. Regulatory mechanisms sustaining these processes have been studied in comparative gene expression analyses between fish and mammals HSC [69], using zebrafish to trigger the functional analysis of mammalian HSC regulator candidates [68,70,71] or even for the screening of biological compounds enhancing HSC production [72,73]. Importantly, these studies in zebrafish contributed to demonstrating the important role of pro-inflammatory signaling in hematopoiesis during normal embryogenesis or in the context of pathogen infections [56,74,75]. In this context, the zebrafish embryo is a valuable model to investigate the impact of infections or IFN on hematopoiesis.

### 3.1. Inflammation, IFNs, and Zebrafish HSC Differentiation in Aseptic Conditions

In homeostatic conditions, the emergence of HSC from the hemogenic endothelium depends on the exposure to inflammatory cytokines and the modulation of conserved transcription factors (*scl*, *lmo2*, *gata1*, *ikaros*, and *spi1*).

In zebrafish, primitive myeloid cells (neutrophils and macrophages) have been identified as major sources of inflammatory cytokines such as TNFα. Primitive myeloid cells are believed to play a major role by producing inflammatory cytokines, thus activating signaling cascade in both endothelial and hemogenic endothelial cells [74] or through direct interactions, as demonstrated by the involvement of macrophages in breaking down the extracellular matrix, allowing the migration of HSC migration and the colonization of hematopoietic organs [76]. TNFα induces jag1a-Notch and NFκB signaling pathways, which are involved in the emergence of HSC [77,78]. Similar to TNFα, type I [78] d type II IFNs [78,79], as well as interleukin IL1β [78] signaling, trigger the emergence of HSC. IFN-γ signaling also acts on the transition from endothelial to HSC, which is a signal transducer and activator of transcription (stat3)-dependent process involving Notch signaling and blood flow [79]. Beside cytokines, miRNA have also been identified as regulators of hematopoiesis. Among the 639 mature miRNA described in zebrafish in the miRBase, miR-126, miR-144, miR-451, and miR-142-3p were respectively involved in erythroid/megakaryocytic lineage, erythropoiesis, and the specification and differentiation of HSC ([80]). The impact of miRNAs on type I or type II IFN responses is still an expanding field.

### 3.2. Impact of Infections and Innate Responses on HSC Differentiation

During infections, pathogen detection rapidly triggers inflammatory responses and hematopoiesis activation, through several non exclusive mechanisms: (1) PAMP sensing by immune cells in hematopoietic sites (2) PAMP sensing in periphery leading proinflammatory cytokines, (3) direct infection of hematopoietic cells and (4) activation through hematopoietic microenvironment [56,74].

These mechanisms were first characterized in the context of bacterial infections in mammalian models. Recruited neutrophils ingest and kill invading bacteria, promoting inflammatory mediator synthesis and secondary recruitment macrophages and professional Antigen Presenting cells (APCs) at the site of infection. The hematopoietic system responds to the increased demand of neutrophils and myeloid cells by switching from steady-state to emergency granulopoiesis [81]. Under Pathogen Associated Molecular Pattern (PAMP) recognition, peripheral infected cells produced IL1β, TNFα, and LPS inflammatory molecules, which induced NFκB and CEBP signaling pathways and increased circulating G-CSF. This directly stimulates the proliferation of granulocytic progenitor cells as receptors for G-CSF, which is mainly expressed in granulocytic precursors and mature neutrophils [82]. The key role of G-CSF in the resolution of bacterial infections has been shown in various mouse models of bacterial infections [82] including *L. monocytogenes* and *Pseudomonas aeruginosa.* Thus, granulopoïesis increases at the expense of lymphoid progenitor cells [83] through the induction of HSPC (hematopoietic stem and progenitor cell) compartment reactivation [84]. Hall et al. [80] recapitulated this “demand-driven” granulopoïesis model in a zebrafish model of infection by *Salmonella*. Following intracerebral injection, the depletion of neutrophils at 1 day post-infection (dpi) is followed by emergency granulopoïesis by 2 dpi at the expense of lymphoid cell progenitors. This was associated with an increased number of HSC at 1 dpi in a detailed CEBP-driven, Nos2a-dependent manner [80]. In a recent work, Willis et al. also demonstrated HSC-driven granulopoïesis in response to *Shigella flexneri* IC injection in zebrafish larvae [85]. Hematopoietic Stem and Progenitor Cell (HSPC) cell death in the context of uncontrolled bacterial burden, and a strong increase of inflammatory cytokine expression (TNFα, IFN 1–2, and IL1) has also been reported [86]. Altogether, these data have established that bacterial infections can increase hematopoiesis via cytokine signaling.

The impact of viral infections on hematopoiesis is less documented, either via direct infection or through IFN-dependent activation. Viral invasions in host organisms face distinct defense mechanisms compared to bacteria, suggesting that their impact on hematopoiesis may rely on different mechanisms. Virus recognition by cellular sensors triggers well-defined signaling pathways and the transcription of pro-inflammatory cytokines, including type I IFN. Then, type I IFN response induced the synthesis of many ISG that count antiviral effector proteins and regulators of immunity. This local and systemic inflammation triggers adaptive immunity and other mechanisms of antiviral defense. Neutrophils, monocytes, and NK cells are recruited for the clearance of deadly infected cells, while lymphocytes contribute to controlling infection in adaptive immunity.

In human, HSPC express Pattern Recognition Receptors (PRR) receptors such as virus-specific Toll-like receptors [87,88] and NOD-like receptors [89]. Their activation leads to HSC cycling and HSPC differentiation, which is preferentially toward mature myeloid cells [87,88,90]. In addition, the direct infection of stem cells, progenitor cells, or stromal cells of a hematopoietic microenvironment have been reported, affecting hematopoiesis via cell death induction or the establishment of viral latency [56]. Systemic acute VSV infection triggers IFN response in bone marrow and HSC, which might evolve in HSC cell cycle entry upon substantial IFN I response. A similar observation was reported in an IFNAR knock-out mouse model with uncontrolled virus replication, which was in favor of an IFNAR-independent HSC activation [91]. Acute infection with murine cytomegalovirus (MCMV) also increased type I IFN expression in HSC and bone marrow (as compared to low systemic levels), and activated HSC through IFNAR-dependent and independent mechanisms. Chronic infection with MCMV was associated with the long-term impairment of long-term HSC [91]. In keeping with this, the chronic infection of human bone marrow progenitor cells has been reported for Human Herpes Virus (HHV)-6, which may be transmitted to differentiated cells, thus using HSC as a source of virus dissemination [92,93]. This was also described for the HHV-8 virus targeting CD34+ hematopoietic stem cells, HHV-7, and HHV-8 infections, leading to the alteration of survival and differentiation of progenitors [92]. However, stem cells have been found to be highly resistant to viral infection across species, due to the intrinsic expression of many ISGs [94]. An additional level of hematopoiesis regulation relies on miRNAs, which are expressed in hematopoietic lineages.

Thus, HSPC fate can be modified by IFN response directly or indirectly via changes in tissue microenvironment stimulation. Several studies have reported the impacts of type I IFN on HSC proliferation, possibly leading to exhaustion through the reduction of quiescent HSCs [95]. In fact, while the chronic activation of the type I IFN in HSCs impairs their function, acute IFNα treatment activates dormant HSC proliferation. However, Poly I:C treatment mediated the return of HSC to quiescence, thus preventing apoptosis and DNA damage [96,97]. The activation of the hematopoietic system by IFN, which is induced after viral infection, has not been extensively studied, although it might be related to several bone marrow pathologies such as aplastic anemia, pancytopenia, hemophagocytic lymphohistiocytosis, lymphoproliferative disorders, and malignancies.

In fish, the modulation of hematopoiesis by viral infection is not well understood either. Viruses targeting hematopoietic precursors have been reported in zebrafish, including Infectious Hematopoietic Necrosis Virus (IHNV) and Infectious Pancreatic Necrosis Virus (IPNV). Infections in adults showed virus persistence over 10 days and cytotoxicity in hematopoietic precursors in the pronephros (the functional equivalent of mammalian bone marrow) with the decrease of terminally differentiated red blood cells, and recovery involving an expansion of proerythroblast [98]. A detailed description of the innate response to IHNV in zebrafish embryos showed that rapid ongoing infection targets endothelial cells. The infection led to 100% mortality between 65–96 Hours post-infection (hpi), which is probably due to late and inefficient host immune response activation; the induction of type I IFN was late from 30 to 48 hpi [99]. In contrast, Chikungunya Virus (CHIKV) replication targets many cell subtypes. The acute phase of infection was associated with an increase in the neutrophils that produce type I IFN, which is critical for survival and probably has an impact on hematopoiesis. Interestingly, this increase is NOS2a-dependent, which is similar to the granulopoiesis induced by Salmonella in [74]. The virus also gains access to the brain, where it persists for longer [100]. Infection of the zebrafish embryo with IHNV or CHIKV leads to different innate responses [101], which may constitute relevant models to evaluate the impact of viral-induced inflammation on hematopoiesis and the priming of adaptive immune responses.

In a model of zebrafish hematopoietic transplant, repeated intra-peritoneal ip injections of the PolyI:C in donors before transplant led to a faster and more efficient colonization of recipient kidney marrow by HSPCs; however, the difference with controls was not significant 28 d post-transplant, and the survival rate was not modified. PolyI:C injections of recipients before transplant did not affect the colonization. While competitive transplants and imaging explorations will be useful to obtain further insights, as proposed by the author, this work shows that valuable in vivo data about the impact of inflammation on HSPC can be produced in zebrafish embryos ([102]; see also https://spiral.imperial.ac.uk/handle/10044/1/55871).

## 4. Coming Insights in Fish Antiviral Immunity from CrispR-Based Genome Editing

Targeted mutation by CRISPR/Cas9-based genome editing is a very efficient methodology to generate knock out genotypes leading to loss of function, which is paramount to the identification of gene function [103]. Although numerous proofs of concepts were established in several fish species by the injection of single guide (sg) RNA and Cas9 into eggs [104], only a few studies have used this technology to investigate the loss of function for genes related to antiviral immunity.

In a first study, *foxo3b^−/−^* mutant zebrafish larvae were generated, and exhibited an increased resistance to infection with spring viremia of carp virus (SVCV), with the expression level of viral genes found to be lower than in the wild-type larvae [105]. In other hands, the over-expression of *foxo3b* reduced the induction levels of ISGs following stimulation with a synthetic dsRNA (poly I:C) or SVCV infection. This clearly indicates the involvement of the *foxo3b* gene in the control of viral infection, and qualifies it as an ISG with inhibitory function. The murine *foxo3b* orthologue counterpart, *foxo3*, was described as part of a negative loop of type I IFN regulation via miR-223 [106]; it was also involved in the control of the T cells’ immune response [106,107]. The latter could be investigated further using the *foxo3b^−/−^* zebrafish in the adult stage.

The second study concerns the farmed rohu carp (*Labeo rohita*). Chakrapani et al. edited the toll-like receptor (*tlr)22* locus using a CRISPR/Cas9-based knock-in approach, but no functional data were reported to confirm the null phenotype in KO fish [108]. TLR22, a dsRNA sensor with slightly different specificity compared to TLR3, is an important component of the viral PAMP repertoire in fish [109,110], and further results are expected from this knock-out study.

Genome editing is more difficult to implement in vitro because it requires the cell line to be able to form single-cell derived clones, which is a condition rarely fulfilled by fish cell lines [111]. Furthermore, the screening of mutant cells is often not a straightforward process, especially in the absence of monoclonal antibodies to demonstrate the disappearance of the targeted protein. However, new sequencing technologies provide powerful solutions both to validate genome editing and characterize resulting phenotypes.

To date, two approaches have been employed to investigate the functions of genes from antiviral pathways in fish cell lines. A first approach is based on the isolation of a salmonid cell line constitutively expressing both the Cas9 and a reporter protein that can be targeted in parallel to the gene of interest [112]. The second approach consists of the use of a plasmid expressing both the Cas9 and the sgRNA targeting the gene of interest [113,114].

**Figure 2:**
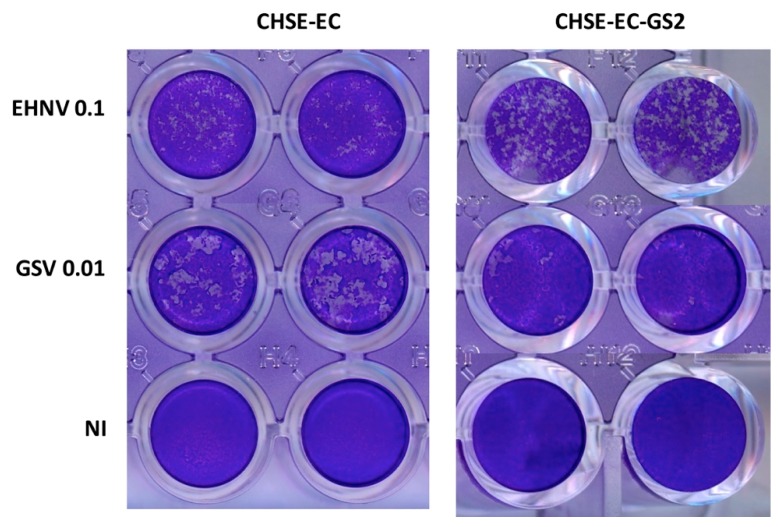
Cytopathic effect of infection with an aquareovirus (golden shiner virus, GSV, MOI = 0.01) or a Ranavirus (epizootic haematopoietic necrosis virus, EHNV, MOI = 0.1) on a salmonid stat2+/+ (EC) or stat2−/− (GS2) cell line. NI: non infected.

Using the first approach, the disruption of several *stat* genes was undertaken in the chinook salmon (*O. tshawytscha)* cell line CHSE. The signaling pathways for type I or II IFN involve two members of the signal transducer and activator of transcription (STAT) family [115]: STAT1 and STAT2. In fish, a variable number of co-orthologs of these genes can be found, because of multiple events of whole genome duplication (WGD). A first WGD occurred during the early evolution of teleost fish about 400 million years ago; more recently (about 100 my ago), a second WGD took place, at the root of the salmonid lineage. As a consequence, a variable number of paralogs with unknown function [116] are typically present in the salmonid genomes. For example, in rainbow trout, coho salmon, and chinook salmon (which all belong to the *Oncorhynchus genus*), there are four *stat1* paralogues. CRISPR/Cas9 genome editing is particularly well suited for the study of such sets of paralogues, as several targets can be edited at the same time within the same cell from which a clonal permanent cell line can be obtained. For example, two out of the four *stat1* paralogues, *stat1a1* and *stat1a2*, were disrupted in a CHSE clone. In these stat1a KO cells, the signaling of both type I and type II IFN was completely obliterated. In contrast, the disruption of the unique *stat2* gene found in the genome affected only type I IFN signaling (Dehler, *et al.* 2019 submitted). Nevertheless, it is remarkable that neither the *stat2^−/−^* cells nor the *stat1a1*^−/−^ *stat1a2*^−/−^ cells showed a drastic increase in viral cytopathogenicity. Furthermore, the dynamic of monolayer destruction appeared to depend on the type of viral pathogen (Figure 2), indicating that this model can help characterize subtle effects of IFN responses on virus/host interactions. Altogether, these results suggest IFN-independent mechanisms of viral resistance similar to those identified in mammals [117,118]. This will be studied in detail by the generation of appropriate knock-out fish cell lines combining the disruption of relevant genes.

Recently, Kim et al. [113] isolated a cyprinid cell line with a disrupted *irf9* gene. The phenotype determined by qPCR gene expression corresponded to a disrupted type I signaling with an obliteration of the induction of ISGs by viral infection or stimulation with dsRNA in the edited cell line compared with the wild type. However, when infected with VHSV, the edited cell line exhibited only a moderate increase in viral titer of less than 1 log. The same group generated a second stable KO model-derived Epithelioma Papulosum Cyprini (EPC), which is a cell line derived from fathead minnow *Pimephales promelas*. The pro-apoptotic gene *hifα*, hypoxia-inducible factor 1-alpha, was disrupted in this KO cell line. Two *hifα* paralogues were found in the fathead minnow genome; the one with higher expression level, *hif1α-ab*, was disrupted. The edited cell line showed a clear decrease in DNA fragmentation after VHSV infection and fewer apoptotic cells after incubation with an apoptosis inducer as visualized in flow cytometry. Unfortunately, comparison of *hif1α-ab−/−* and wild type cells in the absence of infection was not investigated. The edited cell line exhibited a higher resistance to VHSV when compared to the wild type, but with a moderate difference of titer (less than 1 log difference) [114]. Taken together, these results suggest that *hifα* has antiviral effects and controls apoptosis during viral infection. Further work will be needed to fully characterize the consequences of the *hifα* mutation on virus/cell interactions.

Overall, even though both in vivo and in vitro fish knock-out models can be generated, we are still awaiting follow-up studies showing the full potential of these tools. This includes the rigorous genotypic and phenotypic characterization of the KO models with experimental designs that would allow the identification of the functions of antiviral gene candidates with certainty. Although morpholinos are widely used to knock down specific mRNA in zebrafish embryos, genome editing constitutes a critical innovation in many fish models due to the lack of monoclonal Abs directed against key receptors and alternative techniques to manipulate gene expression such as RNA interference. Additionally, CRISPR/Cas-based approaches allow the specific and/or multiple targeting of paralogs. This possibility is critical to dissecting gene sub-functionalization, which is a very common evolutionary pathway for paralogs in families involved in antiviral immunity. Recent developments of CRISPR/Cas protocols have enabled the production of knock-in mutants and punctual genomic mutation. Besides the obvious applications to functional genomics in zebrafish, these approaches offer new perspectives in immunology/genomics in fish species relevant to aquaculture. Thus, the identification of the gene(s) that are responsible for resistance or susceptibility to viral infections is often long and difficult, even when a Quantitative Trait Locus (QTL) has been located [119,120]. Efficient knock-in will constitute perfect solutions to demonstrate the impact of mutations on such complex phenotypes. Altogether, genome editing approaches will empower FAANG ("Functional Annotation of ANimal Genomes") and related initiatives to generate genome-wide functional annotation maps that will be instrumental to exploiting variations in traits of commercial relevance, including resistance to viral diseases.

## 5. Conclusions

Inflammation induced by type I IFN, or independently by viral infection, has critical impacts on the activation of adaptive immunity and hematopoiesis. It also leads to pathologies via multiple signaling pathways that become activated, comprising pro-inflammatory cytokines and fever, NF κB, ubiquitination, proteasome, apoptosis, etc. We believe that fish provide interesting alternative models that can present new insights about these mechanisms, which remain poorly understood. Since it has been demonstrated that the activation of the TLR3 pathway can even facilitate nuclear reprogramming in induced pluripotent stem cells [121], innate antiviral mechanisms appear increasingly important for the control of cell differentiation. The fast development of gene editing technologies that can be used in virtually all species will provide fish immunologists and virologists with exquisite tools to dissect these mechanisms. Besides the interest of highly conserved—and hence likely essential—mechanisms, such studies may also have important impacts through vaccination in aquaculture. Importantly, zebrafish have recently emerged as a promising model for drug screening, and genome editing is likely to provide useful models in this field in the coming years.
